# Frailty Status Moderates the Association Between Self-Efficacy and the Intention to Technology Use

**DOI:** 10.1093/geroni/igab046.2483

**Published:** 2021-12-17

**Authors:** Miseon Kang, Si Young Song, Inhye Jung, YoonMyung Kim, Chang Oh Kim, Hey Jung Jun

**Affiliations:** 1 Yonsei University, Seoul, Seoul-t'ukpyolsi, Republic of Korea; 2 Severance Hospital, Yonsei University College of Medicine, Seoul, Seoul-t'ukpyolsi, Republic of Korea

## Abstract

This study examined how the status of frailty moderated the association between the self-efficacy about gerontechnology use and the intention to use gerontechnology (IUG) among Korean older adults. In this study, gerontechnology devices referred to exoskeleton robots for exercise. The data was collected through an online survey in February 2021, and 324 Korean older adults aged 65 and above were included in the analysis (Women: 50.9%, Men: 49.1%). The dependent variable was the intention to use gerontechnology from the Almere model (Heerink, 2010) and the independent variable was self-efficacy about gerontechnology use from the Senior Technology Acceptance Model(Chen & Chan, 2014). Both were measured as continuous variables. The moderating variable was the status of frailty (Non-frail=0, Frail=1). Age, gender, education level, and log-transformed household income were controlled for. Multiple linear regression to examine moderation effect was conducted using PROCESS Macro model 1. The findings showed that frailty status moderates the association between self-efficacy and IUG among Korean older adults. Concretely, the higher self-efficacy about gerontechnology use, the lower IUG for non-frail Korean older adults. However, the main effect of self-efficacy was non-significant for the frail sample. Even though self-efficacy has been known to affect the variables related to technology use or acceptance positively, the results suggest that there may exist differences in research results depending on participants' health status. The type of gerontechnology devices may also have affected the results. Further exploration is needed to the interaction effects of potential influencing factors on the gerontechnology acceptance model.

